# Abdominal aerobic endurance exercise reveals spot reduction exists: A randomized controlled trial

**DOI:** 10.14814/phy2.15853

**Published:** 2023-11-27

**Authors:** Mathias Forsberg Brobakken, Iben Krogsæter, Jan Helgerud, Eivind Wang, Jan Hoff

**Affiliations:** ^1^ Faculty of Health Sciences and Social Care Molde University College Molde Norway; ^2^ Department of Psychosis and Rehabilitation, Psychiatry Clinic St. Olavs University Hospital Trondheim Norway; ^3^ Department of Circulation and Medical Imaging, Faculty of Medicine and Health Sciences Norwegian University of Science and Technology Trondheim Norway; ^4^ Myworkout, Medical Rehabilitation Clinic Trondheim Norway; ^5^ Department of Physical Medicine and Rehabilitation St. Olavs University Hospital Trondheim Norway

**Keywords:** exercise, selective body fat reduction, subcutaneous fat alteration, upper‐body endurance training

## Abstract

The existence of spot reduction, exercise‐induced local body fat reduction, has been debated for half a century. Although the evidence is equivocal, no study has applied aerobic endurance training closely matching interventions for energy expenditure. Sixteen overweight (BMI: 29.8 ± 3.3(SD) kg m^−2^) males (43 ± 9 years) were randomized to: (1) abdominal endurance exercise (AG), combining treadmill running at 70% HR_max_ (27 min) with 4 × 4 min (30%–40% maximal strength, 1RM) of torso rotation and abdominal crunches (57 min), 4 days⋅week^−1^ for 10 weeks; or (2) control group (CG) performing only treadmill running (45 min) at 70% HR_max_. Local fat mass was measured by dual‐energy x‐ray absorptiometry (DEXA), along with 1RM, and pulmonary oxygen uptake (to control energy expenditure during training). Trunk fat mass decreased more (697 g, 3%, *p* < 0.05) in AG (1170 ± 1093 g, 7%; *p* < 0.05) than in CG (no change). Total fat mass (AG: 1705 ± 1179 g, 6%; CG: 1134 ± 731 g, 5%; both *p* < 0.01) and body weight (AG: 1.2 ± 1.2 kg, 1%, *p* < 0.05; CG: 2.3 ± 0.9 kg, 3%, *p* < 0.01) decreased similarly in AG/CG. Torso rotation (AG: 32 ± 16 kg, 39%, *p* < 0.01; CG: no change) and abdominal crunch 1RM (AG: 35 ± 16 kg, 36%, *p* < 0.01; CG: 13 ± 12 kg, 17%, *p* < 0.05) increased more (*p* < 0.05/0.01) in AG than CG. Abdominal endurance exercise utilized more local fat than treadmill running, indicating that spot reduction exists in adult males.

## 1 INTRODUCTION

Spot reduction, or selective body fat reduction, refers to local subcutaneous adipose tissue loss due to exercise of muscles in the same body segment (Kostek et al., 2007; Ramirez‐Campillo et al., 2013). Although the existence of spot reduction has been a source of debate for over half a century, it has been generally well acknowledged that physical exercise, either involving specific body parts or larger muscle groups, leads to whole‐body adipose tissue utilization rather than subcutaneous fat release stored adjacent to the working muscles (Kostek et al., 2007; Krotkiewski et al., 1979; McArdle et al., 2008a). This commonly held belief, however, is not only based on equivocal data, but also inferred from a body of evidence with experimental considerations which should be more closely addressed.

Strength training, not aerobic endurance training, has predominantly been applied in previous spot reduction studies (Kostek et al., 2007; Krotkiewski et al., 1979; Mohr, 1965; Olson & Edelstein, 1968). This is somewhat surprising since strength training is typically applied, using few repetitions (<15), to increase muscle strength by inducing improvements in neural factors (Toien et al., 2018; Unhjem et al., 2016) or/and muscular factors (Wang et al., 2017). Previous strength training protocols used with the purpose of inducing selective body fat reduction to varying degrees have applied different volumes (repetitions × sets × exercises) and intensities. However, it is unclear if this modality relying on anaerobic energy systems such as high‐energy phosphate bonds or stored muscle glycogen breakdown for fuel (McArdle et al., 2008b) is optimal to evaluate exercise‐induced adipose tissue utilization. Some studies have also applied strength training of relatively small muscle mass (Katch et al., 1984; Kostek et al., 2007), or elected to measure subcutaneous tissue changes with caliper (Gwinup et al., 1971; Kostek et al., 2007; Mohr, 1965; Olson & Edelstein, 1968), which is shown to agree poorly with DEXA and magnetic resonance imaging (MRI; Treuth, Hunter, et al., 1985; Treuth, Ryan, et al., 1985). Others have simply neglected to account for total energy expenditure during training (Carns et al., 1960; Schade et al., 1962). It is thus unclear whether the expended energy would be sufficient to elicit a measurable physiological response, or if the methodology is sensitive enough to be able to detect a change in adipocyte utilization. Despite the fact adipose tissue is the human body's greatest storage of energy, and an essential fuel for metabolism during rest and physical exercise, it is still poorly understood whether exercise may lead to local rather than whole‐body adipose tissue utilization. The inconsistent findings suggest that the concept, if it does exist, remains inadequately described.

To the best of our knowledge, no study has previously examined the distribution of adipose tissue utilization following training by closely matching aerobic endurance exercise interventions for total energy expenditure in a randomized controlled trial. Such a comparison should also involve aerobic endurance training for the abdominal muscles. Thus, in the current study we sought to assess whether a combination of treadmill and abdominal endurance exercises leads to increased trunk adipose tissue utilization beyond what would be expected solely with treadmill exercise. We measured the effects of treadmill and abdominal endurance exercises (torso rotation and abdominal crunches) performed 4 days per week for 10 weeks (aerobic endurance exercise group, AG), on fat mass distribution with DEXA, anthropometry, directly measured maximal oxygen uptake (V̇O_2max_), and maximal strength (one‐repetition maximum, 1RM). The results were compared with a control group (CG) only performing treadmill aerobic endurance training during the study period. Given the general acceptance that spot reduction does not occur, we tested the hypotheses that reductions in fat mass located in the trunk, total fat mass, and body weight would not be different between the AG and CG following the training period.

## 2 MATERIALS AND METHODS

### 2.1 Subjects

**FIGURE 1:**
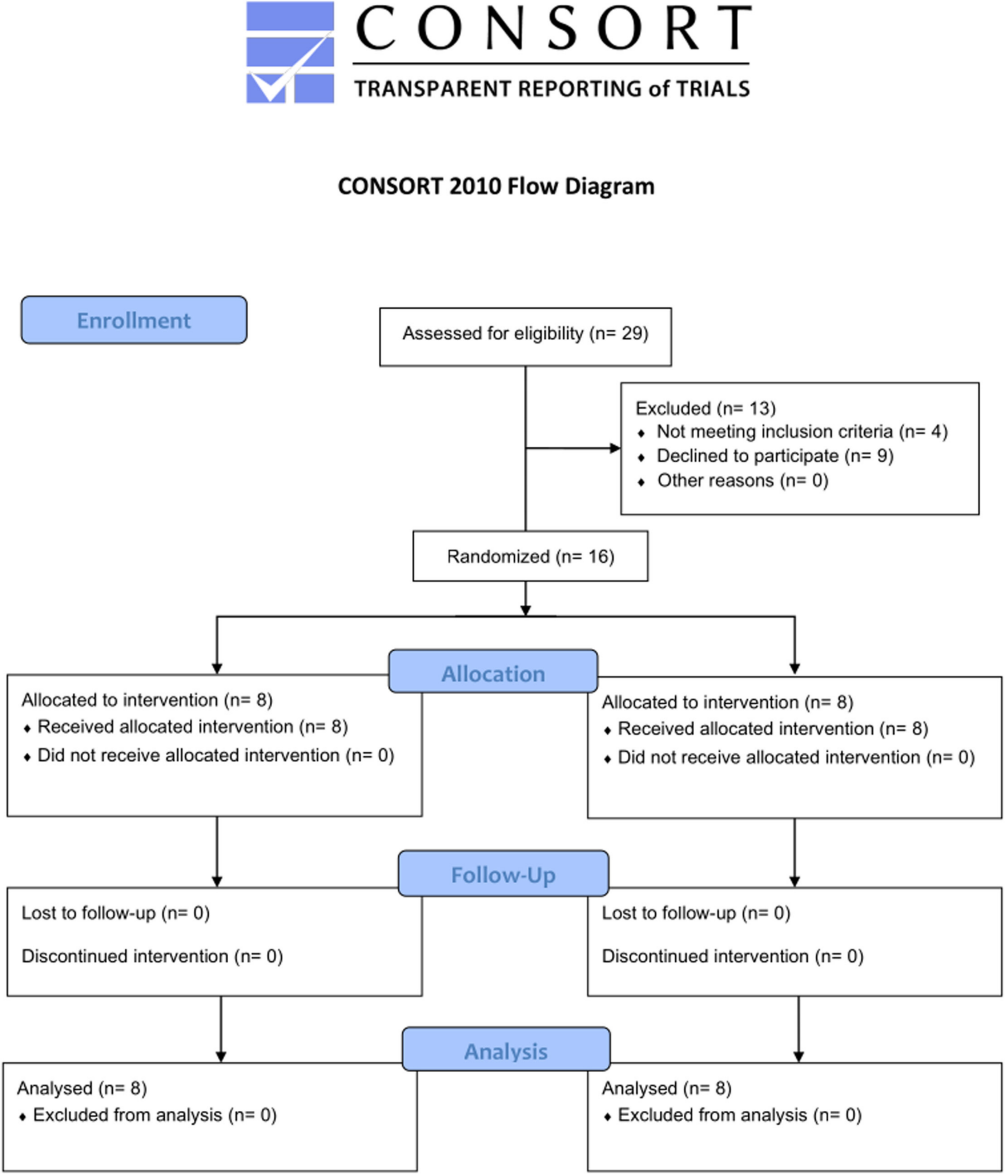
CONSORT flow diagram of the study.

Twenty‐nine male subjects were assessed for eligibility in the current study (Figure 1). Sixteen male subjects (age: 43 ± 9 years; height: 181 ± 7 cm), recruited among students and staff at the Norwegian University of Science and Technology and St. Olavs University Hospital, Trondheim, Norway, volunteered to participate in the study and were randomly divided into one of two groups; the abdominal AG or the CG. Inclusion criteria were males ≥30 years old classified as overweight (body mass index [BMI] ≥ 25 kg m^−2^) who did not currently conduct any form of regular exercise. Written informed consents were collected prior to study inclusion. The study was approved by the institutional review board for medical and health research ethics (Clinicaltrials.gov identifier: NCT05794854), and all procedures were conducted in accordance with the declaration of Helsinki.

### 2.2 Test procedures

Before and after the training intervention, all subjects underwent the following test procedures over three separate days: (1) DEXA and anthropometry, (2) 1RM, and (3) V̇O_2max_.

#### 2.2.1 DEXA and anthropometry

Distribution of fat mass and lean mass in the trunk (abdominal area, chest, and back), lower extremities, and upper extremities were measured with a DEXA device (Discovery A, Hologic, Marlborough, MA, USA) by an independent trained laboratory technician blinded to the subjects' group allocation. DEXA is widely used both for research and clinical settings, providing a precise measure of body composition (Haarbo et al., 1991; Kohrt, 1998). Body composition is measured based on the principle that the intensity of two x‐ray energy beams per pixel relates to the chemical composition, density, and thickness of the different tissues, enabling a discrimination between fat, lean tissue, and bone mass (Hiol et al., 2021). Good accuracy has been documented for the DEXA device with fat mass (0.89%), lean mass (0.76%), and total mass (0.12%) coefficients of variation all <1.0%, suggesting excellent reproducibility (Sutter et al., 2021). Body weight and height were collected by the same exercise physiologist as waist and hip circumference. Waist circumference was measured at navel height at a relaxed standing position, while hip circumference around the widest part of the buttocks, both to the nearest cm.

#### 2.2.2 Maximal muscle strength

1RM tests were carried out in two different abdominal exercise machines: (1) the torso rotation (VR2 Torso Rotation) and (2) the abdominal crunch (VR2 Ab Crunch; both Cybex International, Medway, MA, USA), specifically chosen to target all major abdominal muscles (i.e., m. rectus abdominis, m. obliquus externus/internus abdominis, and m. transversus abdominis). Subjects were instructed to warm‐up by walking/running on a treadmill for 10 min at low to moderate intensity before proceeding to performing 20 repetitions with light loads in the same abdominal exercise machines as used during the test. For both exercises, subjects were told to start in a seated position before commencing the concentric lifting phase. For the torso rotation, both movements to the left and right side were tested, with 1RM collected from the weakest side. During the 1RM test, the load was increased by 2.25–9 kg until the subjects were unable to lift the weight. 1RM was reached within four to six attempts, with each lift interspersed by 2–3 min of rest. The heaviest load a subject could lift represented 1RM.

#### 2.2.3 Maximal oxygen uptake

V̇O_2max_ was pulmonary measured during running on a treadmill (Technogym, Italy) with a Metamax II (Cortex Biophysik, Leipzig, Germany) portable metabolic device. During warm‐up, subjects were instructed to walk/run for 10 min at ~50%–60% of estimated V̇O_2max_. The test proceeded with treadmill velocity and/or inclination being increased by 1 km h^−1^ or 2% each minute until subjects reached exhaustion. Subjects were verbally encouraged throughout the test to elicit maximal effort. V̇O_2max_ was reached within 4–5 min and accepted when V̇O_2_ values reached a plateau despite further increases in treadmill workload and the respiratory exchange ratio was above 1.10 (Wang et al., 2012). Continuous heart rate (HR) measurements were taken during the test with a Polar monitor (Polar Electro, Kempele, Finland). Maximal heart rate (HR_max_) was measured as the highest HR during the test.

### 2.3 Training interventions

The two training interventions utilized in the current study were carefully matched for total energy expenditure. During a pilot study in our laboratory (Table 1), V̇O_2_ and respiratory exchange ratio were measured directly during training sessions in five male subjects (age: 27 ± 2 years, body weight: 76.7 ± 11.4 kg) recruited from students at the University. The same portable metabolic device was used as during the V̇O_2max_ test. To calculate energy expenditure, established thermal equivalents of oxygen for respiratory exchange ratios were used (McArdle et al., 2008c). The pilot study showed that the abdominal endurance exercise yielded 40.4% of the energy expended during one 45‐min treadmill training session. To match interventions for total energy expenditure, additional treadmill running was included in the AG, providing the remaining 59.6% of the energy expenditure. In both groups, training sessions were performed four times per week for 10 weeks with an exercise physiologist as supervisor. All subjects were instructed to abstain from additional exercise training or dietary changes throughout the duration of this study. To be included in analyses, subjects were required to complete ≥75% of the planned sessions in accordance with the protocol.

**TABLE 1:** Pilot testing of oxygen uptake (V̇O_2_) during treadmill and abdominal aerobic endurance exercises.

Subjects	Maximal oxygen uptake, L min^−1^	Measured V̇O_2_, L per session		
		Treadmill^a^	Abdominal aerobic endurance^b^	Difference
1	4.96	121.2	48.9	72.3
2	4.89	120.0	41.1	78.9
3	3.09	74.2	34.5	39.8
4	4.83	116.8	62.9	53.9
5	5.72	142.9	49.6	93.3
Median	4.89	120.0	48.9	72.3

^a^ Treadmill exercise performed for 45 min per session at 70% of maximal heart rate.

^b^ Abdominal crunch and torso rotation performed for a total of 84 min per session, as 4 × 4 min intervals at 30%–40% of maximal strength.

Given that the subjects in the training intervention were older, had higher body weight, and lower V̇O_2max_ than the subjects in the pilot study, we included an additional sample consisting of five male subjects (age: 57 ± 13 years, body weight: 94.9 ± 8.2 kg, BMI: 27.5 ± 1.7 kg m^−2^, V̇O_2max_: 4.0 ± 0.7 L min^−1^; Table S1) with similar characteristics as the subjects included in the AG. Utilizing the same metabolic device and protocol as during the pilot study, V̇O_2max_ was measured along with V̇O_2_ and respiratory exchange ratio during the abdominal endurance exercise and treadmill training session. In this cohort, our data showed that the abdominal endurance exercise required 41.5% of the energy expended during a 45‐min treadmill training session, similar with results from the initial pilot study, indicating that the total energy expenditure was adequately balanced between training interventions.

#### 2.3.1 Abdominal aerobic endurance exercise

Training sessions in the AG started with 27 min of treadmill running at 70% of HR_max_. Subjects proceeded to the abdominal aerobic endurance exercises utilizing the same apparatuses as during the 1RM tests. First, subjects were instructed to undertake 4 × 4 min intervals at 30%–40% of 1RM both directions in the torso rotation machine. The active period on one side was used as resting period for the other. After a 5‐min rest, subjects performed 4 × 4 min intervals at 30%–40% of 1RM in the abdominal crunch machine, interspersed with 3 min active breaks at 20% of 1RM. One training session lasted 84 min in total. To ensure constant rate of performance and control total energy expenditure, a metronome was used and set to 20 repetitions per minute (40 beats min^−1^) for the torso rotation and 40 per minute (80 beats min^−1^) in the abdominal crunch. When subjects were able to complete all abdominal exercise intervals, the load was increased by 2.25 kg and 2.25–4.5 kg in the torso rotation and abdominal crunch exercise, respectively, to maintain the training intensity.

#### 2.3.2 Control group

Subjects randomized to the CG were instructed only to perform treadmill running at 70% of HR_max_ for 45 min. When aerobic endurance capacity improved in the subjects, treadmill velocity and/or inclination were increased to maintain training intensity throughout the study duration. Subjects were not allowed to use the handrails during the treadmill exercise to avoid reducing the total energy expenditure.

### 2.4 Statistical analyses

Statistical analyses were conducted with the software SPSS 12 (IBM SPSS Statistics, Chicago, IL, USA, RRID:SCR_019096) and figures constructed with GraphPad Prism 9 (GraphPad Software, San Diego, CA, USA, RRID:SCR_002798). Data are presented as mean ± SD unless otherwise noted. Non‐parametric tests were utilized to avoid assumption of normal distribution with small groups. Intragroup differences were assessed with Wilcoxon signed rank test, while intergroup differences both at baseline and after the training interventions were examined with Mann–Whitney *U*‐test. A two‐tailed significance level of 0.05 was accepted for all statistical tests.

## 3 RESULTS

The AG and CG, respectively, completed 91 ± 12% and 99 ± 4% of the planned training sessions. All subjects allocated to either training intervention completed ≥75% of the training sessions and were included in the analyses.

### 3.1 Distribution of fat mass, lean mass, and anthropometry

**FIGURE 2:**
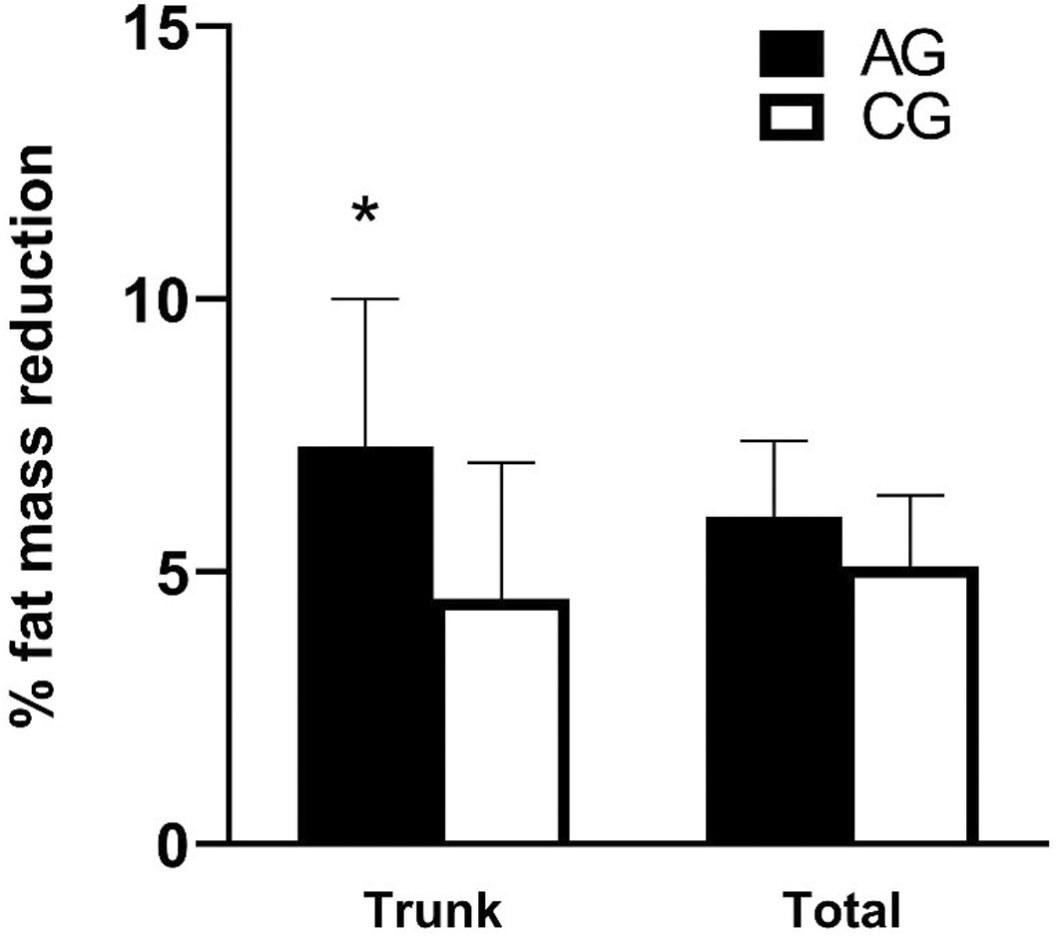
Distribution of fat mass reduction (%) after 10 weeks in the abdominal aerobic endurance exercise group (AG) and treadmill control group (CG). Data are mean ± SE of the mean. *p < 0.05 compared to control group.

No difference between the groups at baseline was observed (Table 2). At baseline, in all included subjects, the trunk, lower extremities, and upper extremities contained 53 ± 4%, 32 ± 4% and 11 ± 1% of the total fat mass, respectively. Trunk fat mass decreased more in the AG (697 g, 3%, *p* < 0.05; Figure 2) than in the CG, where no change was observed. Trunk fat mass was reduced by 1170 ± 1093 g (7 ± 8%, *p* < 0.05) in the AG after 10 weeks, mirrored by a 1.9 ± 1.8% reduction (*p* < 0.05) in percent trunk body fat. Lower extremity fat mass was reduced in both groups (AG: 500 ± 451 g, 6 ± 5%; CG: 507 ± 459 g, 8 ± 8%; both *p* < 0.05), with no intergroup difference observed. This decrease was accompanied by a 0.7 ± 0.8% (*p* < 0.05) lower extremity body fat percentage reduction in the CG. Total fat mass also decreased in both groups (AG: 1705 ± 1179 g, 6 ± 4%, *p* < 0.05; CG: 1134 ± 731 g, 5 ± 4%, *p* < 0.01), again with no difference between groups present. The total fat mass decrement observed in the AG was mirrored by 1.3 ± 0.9% body fat reduction (*p* < 0.05). Total lean mass decreased (1612 ± 1273 g, 2 ± 2%, *p* < 0.05) in the CG after 10 weeks. This decrease was larger (1665 g, 3%, *p* < 0.05) than in the AG, where no change was present.

**TABLE 2:** Distribution of fat mass, lean mass, and anthropometry before and after 10 weeks of abdominal and treadmill aerobic endurance exercise.

	AG (*n* = 8)		CG (*n* = 8)	
	Pre	Post	Pre	Post
Fat mass				
Trunk (g)	16,312 ± 5186	15,141 ± 4965^*,**^	12,185 ± 4790	11,711 ± 4979
% body fat	30.8 ± 5.9	28.9 ± 5.8**	25.9 ± 5.4	25.2 ± 4.7
Lower extremities (g)	9370 ± 2459	8870 ± 2500**	7212 ± 2498	6705 ± 2479**
% body fat	27.1 ± 4.8	26.4 ± 5.2	23.2 ± 2.6	22.6 ± 3.2**
Upper extremities (g)	3124 ± 695	3091 ± 837	2668 ± 1559	2548 ± 1300
% body fat	27.5 ± 4.5	27.4 ± 5.0	23.2 ± 6.2	23.1 ± 6.2
Total (g)	29,946 ± 7763	28,241 ± 7869**	23,156 ± 8923	22,022 ± 8713***
% body fat	28.7 ± 4.7	27.5 ± 4.8**	24.4 ± 4.0	23.8 ± 3.9
Lean mass (g)				
Trunk	34,965 ± 3368	35,508 ± 3153	32,997 ± 3620	32,691 ± 5014
Lower extremities	23,629 ± 1996	23,200 ± 2290	22,216 ± 4367	21,193 ± 3700
Upper extremities	7733 ± 879	7687 ± 1185	7867 ± 1378	7680 ± 1074
Total	70,222 ± 5614	70,275 ± 5628	66,846 ± 9519	65,234 ± 9551*,**
Body weight (kg)	103.1 ± 11.3	101.5 ± 11.7**	92.7 ± 18.3	90.0 ± 18.2***
BMI (kg m^−2^)	30.6 ± 2.2	30.1 ± 2.4**	29.0 ± 4.1	28.1 ± 4.0***
Waist circumference (cm)	113 ± 10	107 ± 9**	103 ± 12	99 ± 12**
Hip circumference (cm)	112 ± 5	110 ± 5	105 ± 5	104 ± 8
Waist‐hip ratio	1.00 ± 0.06	0.97 ± 0.04**	0.98 ± 0.09	0.95 ± 0.06**

*Note*: Data are mean ± SD.*p < 0.05 compared to the other training group; **p < 0.05, ***p < 0.01 from pre‐ to posttest within group.

Abbreviations: AG, abdominal aerobic endurance exercise group; BMI, body mass index; CG, control group.

In both groups, body weight decreased (AG: 1.2 ± 1.2 kg, 1 ± 1%, *p* < 0.05; CG: 2.3 ± 0.9 kg, 3 ± 1%, *p* < 0.01) after 10 weeks, with no difference observed between the groups. This was reflected in reduced body mass index (BMI) in both groups (AG: 0.5 ± 0.5 kg m^−2^, 2 ± 2%, *p* < 0.05; CG: 0.9 ± 0.6 kg m^−2^, 3 ± 2%, *p* < 0.01) following the intervention. Waist circumference also decreased in both groups (AG: 5 ± 3 cm, 5 ± 3%; CG: 5 ± 3 cm, 5 ± 3%; both *p* < 0.05), which was accompanied by reduced waist‐hip ratio (AG: 0.03 ± 0.03; CG: 0.03 ± 0.04; both *p* < 0.05), with no difference between the groups present. Hip circumference remained unchanged in both groups.

### 3.2 Maximal oxygen uptake and maximal muscle strength

In both groups, absolute (AG: 0.26 ± 0.28 L min^−1^, 7 ± 8%; CG: 0.33 ± 0.22 L min^−1^, 8 ± 6%; both *p* < 0.05), relative (AG: 3.1 ± 2.6 mL kg^−1^ min^−1^, 8 ± 8%; CG: 4.9 ± 2.8 mL kg^−1^ min^−1^, 12 ± 6%; both *p* < 0.05), and allometrically scaled (AG: 9.4 ± 8.3 mL kg^−0.75^ min^−1^, 8 ± 8%; CG: 14.1 ± 8.0 mL kg^−0.75^ min^−1^, 11 ± 6%; both *p* < 0.05) V̇O_2max_ increased after 10 weeks, with no intergroup difference present (Table 3).

**TABLE 3:** Maximal oxygen uptake and maximal muscle strength before and after 10 weeks of abdominal and treadmill aerobic endurance exercise.

	AG (*n* = 8)		CG (*n* = 8)	
	Pre	Post	Pre	Post
Treadmill test				
$\dot{V}$O_2max_				
L min^−1^	3.96 ± 0.51	4.22 ± 0.49***	3.92 ± 0.52	4.25 ± 0.53***
mL kg^−1^ min^−1^	39.2 ± 6.9	42.2 ± 6.6***	43.9 ± 8.3	48.9 ± 9.0***
mL kg^−0.75^ min^−1^	124.0 ± 19.4	133.4 ± 18.4***	134.8 ± 22.6	148.9 ± 24.0***
HR_max_, beats min^−1^	184 ± 12	181 ± 10***	187 ± 15	184 ± 12
One‐repetition maximum (kg)				
Torso rotation	90 ± 22	123 ± 24^*,****^	88 ± 22	96 ± 23
Abdominal crunch	106 ± 21	141 ± 23^**,****^	111 ± 27	125 ± 19***

*Note*: Data are mean ± SD.*p < 0.05, **p < 0.01 compared to control group; ***p < 0.05, ****p < 0.01 from pre‐ to posttest within group.

Abbreviations: AG, abdominal aerobic endurance exercise group; CG, control group; HR_max_, maximal heart rate; $\dot{V}$O_2max_, maximal oxygen uptake.

Torso rotation 1RM increased more (25 kg, 27%, *p* < 0.05) in the AG (32 ± 16 kg, 39 ± 23%, *p* < 0.01) compared to the CG where no change was present. Abdominal crunch 1RM also increased more (22 kg, 20%, *p* < 0.01) in the AG (35 ± 16 kg, 36 ± 20%, *p* < 0.01) compared to the CG (13 ± 12 kg, 17 ± 16%, *p* < 0.05).

## 4 DISCUSSION

It has been well accepted that exercise‐induced lipolysis occurs predominantly through whole‐body fat release, rather than from local adipose tissue storages adjacent to the working muscles. However, to date no study has investigated the concept of spot reduction/lipolysis by comparing abdominal aerobic endurance training (AG) with whole‐body treadmill endurance training (CG) closely matched for total energy expenditure in a randomized controlled trial. The main finding from the current study was that after 10 weeks and 40 training sessions, fat mass located in the trunk decreased more (697 g, 3%) in the AG than in the CG, while total fat mass and body weight decreased similarly in both groups. These findings demonstrate the efficacy of the protocol to investigate differences in adipose tissue utilization, and that spot reduction exist in the trunk region in overweight adult males.

### 4.1 Spot versus generalized fat reduction during abdominal aerobic endurance training

In contrast to our hypothesis, the current study found that fat mass stored in the trunk decreased more in the AG than in the CG. In fact, intragroup analyses showed that trunk fat mass decreased by 1170 g (7%) in the AG, with no detectable change observed in the CG. To the best of our knowledge, our study is the first to document that aerobic endurance exercise of the abdominal body segment leads to increased release and utilization of adipose tissue stored adjacent to the working muscles, rather than generalized whole‐body fat release.

Our results contrast with previous observations by Ramirez‐Campillo et al. (2013) who failed to observe a change in DEXA‐measured local fat mass in 11 men and women following 12‐weeks of one‐legged endurance training, performed at 10%–30% of 1RM for 80 min per session, with the untrained leg serving as the control condition, following their 12‐week training intervention. Although our studies are certainly similar in terms of number (36 vs. 40) and duration (80 vs. 84 min) of the training sessions, it may be that our choice of abdominal rather than lower extremity exercises, applied only in men, is the key to understand the different findings. It is shown that upper rather than lower body fat depots are to greater extent mobilized for mitochondrial oxidation during moderate intensity exercise (Horowitz, 2003). This may be caused by factors such as higher hormone sensitive lipase activation, ratio of beta/alpha‐adrenoceptor density and affinity, and subcutaneous adipose tissue blood flow, most likely augmenting the propensity for fat metabolism in favor of the trunk compared to the lower extremities (Tan et al., 2004; Wahrenberg et al., 1989). Our inclusion of men may provide further explanation of our data, given that regional differences (between abdominal and gluteal sites) in catecholamine‐induced lipolysis also appear to be even more pronounced in women, particularly through the lipolysis‐limiting effect of higher alpha 2‐adrenergic receptor affinity (Wahrenberg et al., 1989). Since plasma concentrations of catecholamines are shown to increase proportionally with endurance exercise intensity (Romijn et al., 1993), the somewhat higher intensity applied in the current study, that is, 30%–40% of 1RM, may also have impacted results.

Our findings also contrast with the seminal work by Noland and Kearney (Noland & Kearney, 1978), who reported that general aerobic and localized exercises both resulted in reduced caliper‐measured skinfolds and girths at the umbilical level and iliac crest in women, albeit with little or no intergroup difference. Likely contributing to the lack of difference in adipose tissue alterations, the endurance exercises applied in both groups, although described as more typical aerobic versus calisthenic‐type activities, targeted predominantly the same major muscle groups. With little control of energy expenditure other than sporadic heart rate recordings, these factors taken together would unlikely yield significantly different intergroup results even with state‐of‐the‐art DEXA or MRI recordings (Noland & Kearney, 1978).

### 4.2 Spot reduction, intensity, and metabolic versus circulatory demands

It remains unclear whether the effects observed in the current study are predominantly related to metabolic or circulatory mechanisms during training or recovery after exercise. The rate of peripheral adipocyte lipolysis, from subcutaneous or visceral adipose tissue, is documented to be highest during exercise with low intensity and decreases as intensity progresses (Romijn et al., 1993). In contrast, lipolysis of intramuscular triglycerides is augmented with increasing intensity (Romijn et al., 1993). As such, it is possible that these energy stores, beneficially located in near proximity to the working muscles rather than transported via the blood stream, contribute to the metabolism during training. The somewhat smaller muscle mass involved in the abdominal exercises, as opposed to whole‐body endurance training, may lead to higher mass‐specific rate of fat oxidation than during large muscle mass exercise, possibly due to increased perfusion and favorable O_2_ delivery conditions (Skattebo et al., 2022). However, the relatively high training intensity indicates a greater reliance on carbohydrate as the substrate of choice during exercise (Romijn et al., 1993), as fat is a significantly less O_2_‐efficient fuel, yielding ~4.6 mole ATP per mole O_2_ consumed compared to ~5 mole for carbohydrate (Spriet, 2014). As such, the combination of high intensity and increased trunk fat mass mobilization after abdominal endurance training may suggest that fat depots in close proximity, such as intramuscular triglycerides, are mobilized to replenish glycogen storages through gluconeogenetic pathways after training.

In theory, if spot reduction was mainly caused by replenishment of glycogen storages through gluconeogenetic processes during recovery (Frayn, 1983), one would expect that strength training, performed with high volume and long duration resulting in depleted glycogen storages of the trained muscles, would lead to a measurable physiological response. This may be part of the explanation, given that findings from studies applying strength training, with varying degrees of volume, to examine this concept vary quite considerably. While some strength training studies utilizing intraindividual designs suggest spot reduction may occur (Mohr, 1965; Olson & Edelstein, 1968), possibly due to reduced fat cell size (Katch et al., 1984; Krotkiewski et al., 1979), others have rebuffed this notion (Kostek et al., 2007).

In the CG, total lean mass decreased by 1.6 kg (2%) after 10 weeks, more than in the AG where no change was observed. As basal metabolic rate is closely related to changes in fat‐free mass, a 1.6 kg decrease in lean tissue may result in reduced energy expenditure by ~25–35 kCal per day, and in turn less fat oxidation (McArdle et al., 2008d; Ravussin et al., 1986). However, considering that the V̇O_2max_ in the CG increased throughout the intervention, a decline in daily energy expenditure would likely be offset by a greater increase in energy expenditure during treadmill endurance training. As a result, the relatively small reduction in lean tissue mass would unlikely lead to less decrease in trunk fat mass than in the AG during the study period.

The intensity applied in the abdominal endurance exercises for the current study, chosen specifically to emulate aerobic interval training, is shown to improve oxygen transport and utilization (Helgerud et al., 2007, 2009; Liu et al., 2022). Aerobic endurance training with the high intensity format 4 × 4 min intervals may be applied to both whole‐body and small muscle mass exercise, and is excellent for overloading the involved oxygen transporting organs (Helgerud et al., 2007, 2009). Indeed, high intensity (> 85% of maximal heart rate) is documented to improve skeletal muscle capillarization, expressed as capillary to fiber ratio and capillary density, substantially more than low or moderate intensity endurance training (Liu et al., 2022). Another mechanism potentially explaining spot reduction may be related to improved circulation in subcutaneous adipose tissue. Notably, even after only an acute bout of endurance exercise, mRNA expression of VEGFA, an important regulator of angiogenesis and capillary growth in subcutaneous adipose tissue, is shown to increase in overweight and obese adults (Van Pelt et al., 1985). Although the scarce evidence warrants caution, it is plausible that improved circulatory adaptations following endurance training would produce more favorable conditions for release and transportation of body fat. Taken together, this may suggest that the observed increase in trunk adipose tissue utilization following abdominal endurance training may be, at least in part, related to improved blood flow kinetics and circulatory mechanisms in the trained muscle bed and/or subcutaneous adipose tissue.

### 4.3 Clinical implications and limitations

Each full training session in the AG, combining treadmill and abdominal aerobic endurance exercise, lasted in total 84 min, almost double the duration compared to the moderate intensity treadmill intervention in the CG. The smaller muscle mass used in the abdominal endurance exercise, as compared to whole‐body treadmill exercise, necessitates longer duration to match for total energy expenditure. Although the subjects were instructed not to change their dietary habits during this study, including a measure of dietary control would have strengthened the study design, as energy balance, that is, energy intake versus expenditure, impacts whether excess energy is stored as fat or used as fuel. While we did not observe a significant difference in body composition between the training groups at baseline, we cannot fully discount the possibility that the somewhat higher body fat and lean tissue mass in the AG increased the availability of fat to be utilized during training. However, no difference in total fat mass reduction between the groups was observed. This suggests not only that the external work output, and therefore energy requirement, was adequately balanced between the interventions, but also that it is unlikely any numeric difference in body composition at baseline would have influenced the amount of local adipose tissue loss to any large extent.

The fact that we did not observe a difference in the percentage energy cost of the abdominal aerobic endurance training compared to treadmill training between our pilot test (40.4%) and supplementary test subjects (41.5%), and the use of relative workloads (% of HR_max_ and 1RM) should be regarded as strengths of the study. However, an individual rather than group‐based equalization between training protocols may have improved the design further. Although the DEXA provides valid and accurate measures of regional fat mass and lean tissue based on tissue density/thickness, data differentiating between visceral, subcutaneous, and intramuscular adipose tissue is lacking, thus it is unclear which fat depots are mobilized during this training intervention. As described, sex differences in lipolytic mechanisms exist, and as such it is unclear if the findings translate to women. The current model, utilizing an endurance training protocol in the abdominal region, should also be replicated in other body segments to improve our understanding of potential regional differences in spot reduction.

## 5 CONCLUSIONS

The current randomized controlled trial provides evidence that aerobic endurance training of a specific body segment may in fact lead to increased utilization of adipose tissue locally stored. Specifically, our data highlight that 10 weeks of aerobic endurance training of the abdominal region, with relatively high intensity and matched for total energy expenditure, increased adipose tissue utilization from the trunk more than the whole‐body treadmill endurance training performed in the CG. In conclusion, our data advocate that spot reduction exists in overweight adult males.

## AUTHOR CONTRIBUTIONS

Jan Helgerud and Jan Hoff conceived and designed the study. Iben Krogsæter conducted the experiments. Iben Krogsæter, Jan Hoff, Eivind Wang, and Mathias Forsberg Brobakken analyzed the data. Mathias Forsberg Brobakken and Eivind Wang wrote the manuscript. All authors critically read, revised, and approved the manuscript.

## CONFLICT OF INTEREST STATEMENT

None.

## ETHICS STATEMENT

The study was approved by the institutional review board for medical and health research ethics (Clinicaltrials.gov identifier: NCT05794854), and all procedures were conducted in accordance with the declaration of Helsinki.

## Supporting information


Data S1.

## Data Availability

The data that support the findings of this study are available from the corresponding author upon reasonable request.
