# Supplementary material for: Abdominal aerobic endurance exercise reveals spot reduction exists: A randomized controlled trial
Source: Physiol Rep. 2023 Nov 27;11(22):e15853. doi: 10.14814/phy2.15853 (PMC10680576; doi:10.14814/phy2.15853)
Supplement: Supplementary file 1 — Data S1. [file PHY2-11-e15853-s001.docx]

**Supplementary table.** Oxygen uptake (V̇O_2_) during treadmill and abdominal aerobic endurance exercises of additional included subjects representative of the training intervention sample

| Subjects | Maximal oxygen uptake,  L ⋅ min^-1^ | Measured V̇O_2_, L per session | | |
| --- | --- | --- | --- | --- |
|  |  | Treadmill ^a^ | Abdominal aerobic endurance ^b^ | Difference |
| 1 | 4.00 | 97.2 | 39.4 | 57.8 |
| 2 | 4.56 | 108.8 | 44.7 | 64.1 |
| 3 | 5.17 | 118.7 | 52.7 | 66.0 |
| 4 | 3.28 | 95.9 | 41.2 | 54.7 |
| 5 | 3.99 | 107.7 | 48.5 | 59.2 |
| Median | 4.00 | 107.7 | 44.7 | 59.2 |

^a^ Treadmill exercise performed for 45 minutes per session at 70 % of maximal heart rate; ^b^ abdominal crunch and torso rotation performed for a total of 84 min per session, as 4 x 4 minutes intervals at 30-40 % of maximal strength.
